# Preliminary Molecular Characterizations of *Sarcoptes scaibiei* (Acari: Sarcoptidae) from Farm Animals in Egypt

**DOI:** 10.1371/journal.pone.0094705

**Published:** 2014-04-11

**Authors:** Said Amer, Taher Abd El Wahab, Abd El Naby Metwaly, Jianbin Ye, Dawn Roellig, Yaoyu Feng, Lihua Xiao

**Affiliations:** 1 Division of Foodborne, Waterborne and Environmental Diseases, Centers for Disease Control and Prevention, Atlanta, Georgia, United States of America; 2 Department of Zoology, Kafr El Sheikh University, Kafr El Sheikh, Egypt; 3 Animal Health Research Institute, Kafr El Sheikh Provincial Laboratory, Kafr El Sheikh, Egypt; 4 State Environmental Protection Key Laboratory of Environmental Risk Assessment and Control on Chemical Process, East China University of Science and Technology, Shanghai, People's Republic of China; University of the Sunshine Coast, Australia

## Abstract

Little is known about the genetic diversity of *Sarcoptes scabiei* mites in farm animals in Egypt. In this study, we characterized *S. scabiei* in 25 skin scrapes from water buffalo, cattle, sheep, and rabbits at the nuclear marker ITS2 and mitochondrial markers COX1 and 16S rRNA. Sequences of the ITS2 showed no host segregation or geographical isolation, whereas those of the mitochondrial COX1 and 16S rRNA genes indicated the presence of both host-adapted and geographically segregated populations of *S*. *scabiei*. Host adaptation may limit inter-species transmission of. *S. scabiei*, thus restrict gene flow among *S. scabiei* from different hosts. This is the first report on the molecular characterization of sarcoptic mites in Egypt. Further genetic studies involving larger numbers of specimens, especially those from humans and companion animals, are needed to understand the molecular epidemiology of sarcoptic mange in Egypt.

## Introduction

The cosmopolitan mite *Sarcoptes scabiei* (Acari: Sarcoptidae) is an obligatory ectoparasite that infects the skin of a wide range of mammalian hosts, resulting in sarcoptic mange in companion animals, livestock, and wildlife, as well as scabies in humans [1]–[3]. The disease is highly contagious, characterized by pruritic dermatitis, alopecia, hyperkeratosis, and crust formation [4]–[6], and if left untreated, can lead to death due to dehydration, pneumonia, or bacterial septicemia [7]–[9]. In addition to its potential to cause huge economic losses due to weight loss and mortality in animals [10], [11], scabies imposes a global public health concerns as an emerging/re-emerging infectious disease [9], [12], [13]. Scabies outbreaks have been reported in industrialized countries [14]–[16], and the burden of the disease in developing countries is increasing [1], [17]–[19]. Drug residuals and toxicity due to extensive use of acaricides, especially in developing countries, and emergence of drug resistance are some other growing problems associated with sarcoptic mange and scabies [20]–[23].

Current knowledge suggests that humans and protohumans were most likely the initial source of animal scabies, first of dogs, and later of other species with further spread to wildlife [16]. *Sarcoptes scabiei* is taxonomically divided into different varieties based on host origin [24]. However, speciation in *S. scabiei* is a controversial issue due to the indistinguishable morphology of host-associated populations, evidence of apparent cross-species transmission during epizootics in sympatric wild animals [12], [25], limited or no cross-infestations between hosts in experimental studies [26], and presence of immunologically host-specific and cross-reactive epitopes [27], [28]. Characterizations of mitochondrial DNA (mtDNA) haplotypes and microsatellite allele frequencies have demonstrated significant associations between *S. scabiei* and host species or geographical locations [29], [30].

In Egypt, scabies has been reported in farm animals [31]–[33], wild games [34], and human [35]–[38]. However, there are no data on genetic diversity of *S. scabiei*. This preliminary study was conducted to examine the genetic characteristics of *S. scabiei* derived from different hosts in Egypt, including water buffalo, sheep, rabbits, and one cattle. Results of sequence characterization of the nuclear internal transcribed spacer 2 (ITS2) and mitochondrial cytochrome oxidase 1 (COX1) and 16S rRNA genes demonstrated the presence of host-adapted and geographically segregated *S. scabiei* populations in Egypt.

## Materials and Methods

### Ethics Statement

This study was carried out in strict compliance with the Guidelines of Animals Health Research Institute, Egypt. The study protocol was approved by the Committee on the Ethics of Animals Health Research Institute, Egypt (Permit Number 362 approved on August 31, 2010). All scrapings were collected by well trained and licensed veterinarians. This study was done on specimens from animals on private farms as part of the routine clinical examinations and care, with written consents from the owners. One of the co-investigator of the project, Dr. Abd El Naby Metwaly (Animal Health Research Institute, Kafr El Sheikh Provincial Lab, Kafr El Sheikh 33516, Egypt; e-mail: tahoon63@yahoo.com), should be contacted for permissions for future work on these farms. Efforts were made to minimize discomfort and stress to animals while performing skin scraping.

### Specimens

Specimens of this study were collected during August 2010-April 2011 from buffalo, sheep, rabbits, and one cattle (Table 1) in Sheikh Province (130 km north of Cairo). Buffalo specimens were collected from animals on three farms at kafr El Sheikh District; specimens that had mixed infection with *Psoroptes* spp at the same infection site were excluded. Rabbit specimens were collected from three small rabbitries; rabbits were raised in wired cages. Sheep specimens were collected from two farms, with goats raised in the same herd on the second farm. The cattle specimen was from a sporadic case for veterinary consultation. Skin scrapings were collected in situ directly from infected animals into tightly closed plastic cups, transferred to the laboratory, and examined by microscopy. Positive samples were fixed in 75% ethyl alcohol and stored at 4°C for molecular biologic analyses.

**Table 1:** *Sarcoptes scabiei* isolates collected from four species of farm animals at Kafr El Sheikh Province, Egypt.

Specimen ID	Host	Farm	Infection Site
37025	Water buffalo	Buffalo farm 1	Perineal region
37026	Water buffalo	Buffalo farm 1	Perineal region
37027	Water buffalo	Buffalo farm 1	Perineal region
37028	Water buffalo	Buffalo farm 1	Perineal region
37031	Water buffalo	Buffalo farm 2	Perineal region
37032	Water buffalo	Buffalo farm 2	Perineal region
37033	Water buffalo	Buffalo farm 2	Perineal region
37030	Water buffalo	Buffalo farm 3	Perineal region
37073	Cattle	Sporadic case	Body
37061	Sheep	Sheep farm 1	Body
37077	Sheep	Sheep farm 1	Body
37078	Sheep	Sheep farm 2	Body
37080	Sheep	Sheep farm 2	Body
37081	Sheep	Sheep farm 2	Body
37040	Rabbit	Rabbitry 1	Body
37053	Rabbit	Rabbitry 1	Ear
37054	Rabbit	Rabbitry1	Ear
37056	Rabbit	Rabbitry 1	Ear
37059	Rabbit	Rabbitry1	Foot
37065	Rabbit	Rabbitry 1	Body
37060	Rabbit	Rabbitry 2	Foot
37063	Rabbit	Rabbitry 2	Ear
37074	Rabbit	Rabbitry 2	Ear
37064	Rabbit	Rabbitry 3	Foot
37075	Rabbit	Rabbitry 3	Ear

### DNA extraction and PCR analysis

Ethyl alcohol was removed from microscopy-positive specimens by centrifugation and washing with distilled water. DNA was extracted from the specimens using the FastDNA SPIN Kit for Soil (MP Biomedicals, Colon, OH). PCR amplification of the ITS-2 was done using primers RIB-18 and RIB-3 as described by Zahler et al. [39]. PCR analyses of the mitochondrial COX1 and 16S rRNA genes were conducted as described by Walton et al. [30].

### DNA sequence analyses

PCR products were sequenced directly using Big Dye Terminator v3.1 Cycle Sequencing Kit (Applied Biosystems, Foster City, CA) and an ABI 3130 Genetic Analyzer (Applied Biosystems). Sequences were assembled using the ChromasPro (version 1.5) software (http://www.technelysium.com.au/ChromasPro.html). The accuracy of data was confirmed by bi-directional sequencing. The obtained sequences were aligned with each other and reference sequences of each gene using ClustalX (ftp://ftp-igbmc.u-trasbg.fr/pub/ClustalX/) to confirm the identification of *S. scabiei*. A neighbor-joining (NJ) analysis implemented in the MEGA5 (http://www.megasoftware.net) was used to assess the phylogenetic relationship among different populations of *S. scabiei*. Unique nucleotide sequences generated in this study were deposited in GenBank under accession numbers AB778895 to AB778919 for ITS2, AB779564 to AB779587 for mitochondrial 16S rRNA, and AB779588 to AB779611 for mitochondrial COX1 genes.

## Results

**Figure 1:**
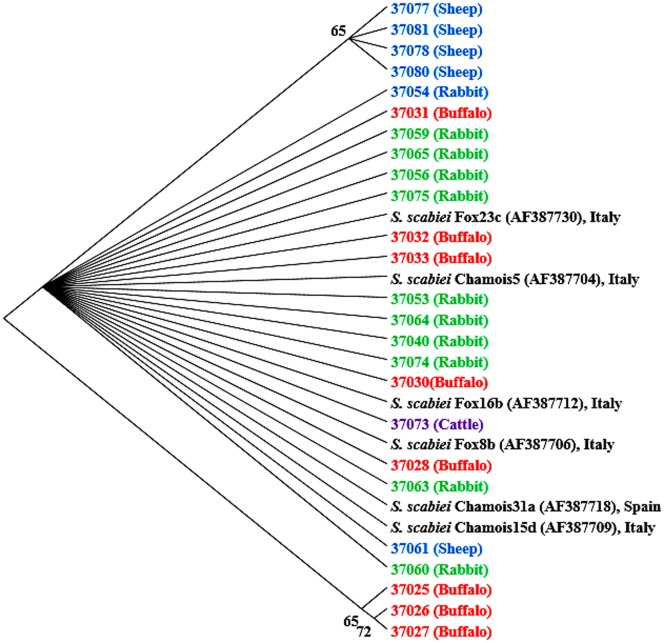
Un-rooted NJ tree showing genetic relationship of Egyptian *Sarcoptes* mites to others in the GenBank database based on ITS2 sequences. Evolutionary relationships of 31 taxa were inferred using the neighbor-joining method [46]. Numbers at the internodes correspond to percent bootstrap values from 2,000 replicates. Branches corresponding to partitions produced in less than 50% bootstrap replicates are collapsed. Sequences in bolded colors and with no GenBank accession numbers are generated from Egyptian specimens.

ITS2 sequence analysis of the *Sarcoptes* mites derived from different hosts from Egypt generated 7 sequence types. Despite the low number of polymorphic sites (5 sites), these sequences formed three groups on the NJ tree. All sequences derived from mites in rabbits, several sequences from mites in buffalo, and one sequence each from mites in cattle and sheep formed a cluster on the tree together with reference sequences from GenBank (Fig. 1), reflecting the broad host and geographical distribution of this *S. scabiei* population. In contrast, the other two groups were formed by sequences from this study, including one group containing the majority of sequences from mites of sheep (37077, 37078, 37080 and 37081), and one containing 3 sequences (37025–37027) from mites of buffalo.

**Figure 2:**
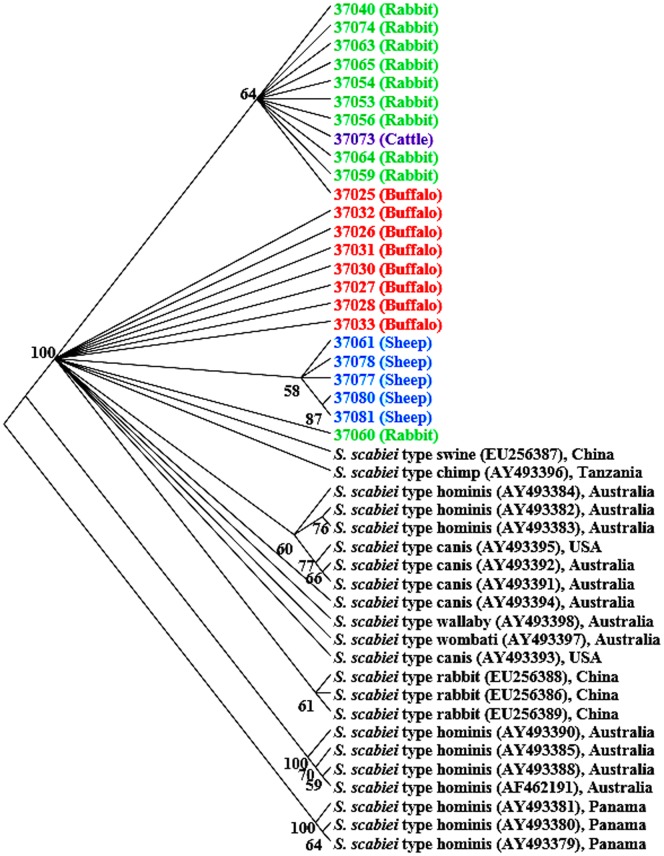
Un-rooted NJ tree showing genetic relationship of Egyptian *Sarcoptes* mites to others in the GenBank database based on COX1 sequences. Evolutionary relationships of 46 taxa were inferred using the neighbor-joining method [46]. Numbers at the internodes correspond to percent bootstrap values from 2,000 replicates. Branches corresponding to partitions produced in less than 50% bootstrap replicates are collapsed. Sequences in bolded colors and with no GenBank accession numbers are generated from Egyptian specimens.

Sequence variability was greater at the mitochondrial COX1 and 16S rRNA genes. Altogether, 10 types of COX1 sequences and 5 types of 16S rRNA sequences were obtained, which differed from each other in the form of nucleotide substitutions and insertions or deletions. NJ analysis based on COX1 clearly showed the presence of 2 major clusters of *S. scabiei* in Egypt by host (Fig. 2). One cluster included all sequences from mites in rabbits, one cattle (37073), and one buffalo (37025). The other cluster had two branches, one of *S. scabiei* in sheep and one of *S. scabiei* in buffalo (Fig. 2). Comparing to *S. scabiei* isolates from other areas, the Egypt-derived sequences occupied unique positions in the NJ tree (Fig. 2). Phylogenetically, COX1 sequences from human isolates in Panama and some human isolates in Australia formed the two basal branches diverged from others containing sequences mostly from animal isolates. In the latter branches, sequences from different animals in different geographical locations showed host and geographical clustering, with sequences from Egyptian isolates separated from others. Sequences from several other human isolates in Australia formed a subgroup within the major cluster of largely animal isolates (Fig 2).

**Figure 3:**
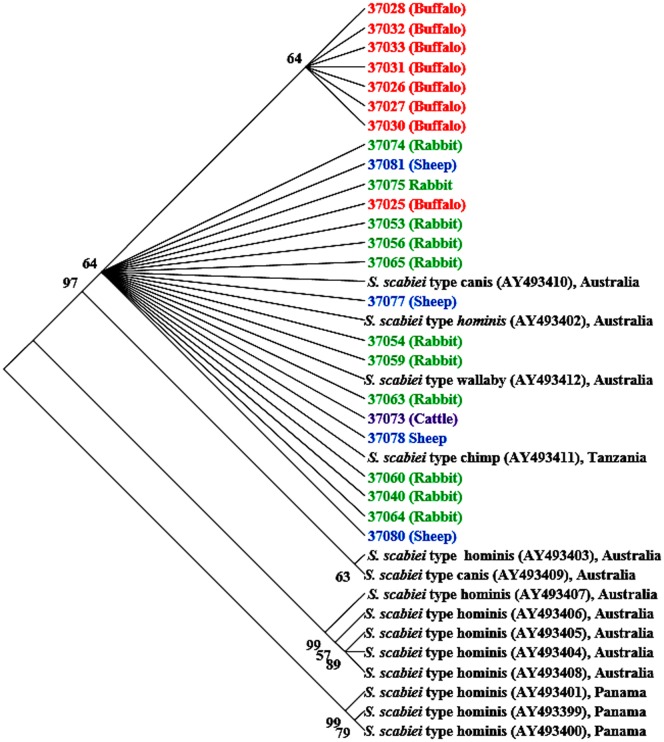
Un-rooted NJ tree of showing genetic relationship of Egyptian *Sarcoptes* mites to others in the GenBank database based on mitochondrial 16S rRNA sequences. Evolutionary relationships of 38 taxa were inferred using the neighbor-joining [46]. Numbers at the internodes correspond to percent bootstrap values from 2,000 replicates. Branches corresponding to partitions produced in less than 50% bootstrap replicates are collapsed. Sequences in bolded colors and with no GenBank accession numbers are generated from Egyptian specimens.

Sequences of the mitochondrial 16S rRNA gene divided the Egyptian *S. scabiei* isolates into two major groups, one containing most buffalo isolates and one containing all sheep and rabbit isolates and one each of buffalo and cattle isolates. In concordance with results of the COX1 sequence analysis, a NJ tree based on the 16S rRNA gene sequences placed sequences from all human isolates in Panama and some human isolates in Australia in the basal branches divergent from sequences from most animal isolates in various areas and some human isolates in Australia. However, there was less host and geographic segregation in the latter groups than seen at the COX1 locus, although the sequences from buffalo in Egypt clearly formed its own clade (Fig. 3). Sequences from isolates in rabbits, sheep, and one buffalo and cattle each were more related to each other and clustered together with sequences AY493410 from a dog in Australia, AY493402 from a human in Australia, AY493412 from a wallaby in Australia, and AY493411 from a chimpanzee in Tanzania. Thus, *S. scabiei* from sheep was genetically related to *S. scabiei* from buffalo at the COX locus, but was more related to *S. scabiei* from rabbits at the 16S rRNA locus, even though both loci are in the small mitochondrial genome. The relatively low bootstrap values of most branches in the phylogenetic trees were probably the result of limited sequence polymorphism and random distribution of some nucleotide substitutions at these loci.

## Discussion

In this study, we sequence-characterized *S. scabiei* isolates from buffalo, sheep, rabbits, and one cattle at three genetic loci. Results obtained showed that ITS2 sequences from *Sarcoptes* mites from these hosts are conserved with intra-sequence variability at only 5 positions. These results are in concordance with those of Zahler et al. [39] and Gu and Yang [40]. Thus, based on ITS2 sequence analysis, Zahler et al. [39] reported very little genetic variation in sarcoptic mites collected from different hosts and geographic locations, and, Gu and Yang [40] could not differentiate *Sarcoptes* mites from different hosts in China. Although Berrilli et al. [41] and Alasaad et al. [25] detected some gene variability between individual mites, the sequence variations were randomly distributed in different hosts from several locations, thus resulting in no distinct geographic or host-specific clustering.

In contrast to the nuclear ITS2 marker, mitochondrial markers analyzed in the present study showed clear sequence polymorphism related to the host species. COX1 sequence analysis showed the presence of 3 distinct groups by host species with additional geographic stratifications (Fig. 2). This was also supported by results of sequence analysis of the mitochondrial 16S rRNA gene (Fig 3). Using hypervariable microsatellite loci, Walton et al. [42] reported that *S. scabiei* from dogs and humans clustered by host species rather than by geographic location. In contrast, phylogenetic studies based on 16S rRNA and COX1 sequences demonstrated that clustering patterns of S. *scabiei* mites were under the impact of both host species and geographical locations [30], whereas sequence analysis of the mitochondrial 12S rRNA gene did not show any significant association between haplotypes and host species [43]. Thus, multilocus characterization of diverse isolates is needed to better understand host adaptation and geographic segregation in *S. scabiei*.

Host adaptation in *S. scabiei* has important implications in understanding the epidemiology and development of diagnostic tests and vaccines [30]. Previously, it was thought there was frequent inter-breeding among mites infecting distinct host species, increasing their genetic variability and allowing *Sarcoptes* to infect new species of animals [44]. In contrast, results of recent studies have shown the occurrence of host adaptation in *S. scabiei* [30], [42]. Data of the present study indicate that both host adaptation and geographic segregation also occur in *S. scabiei* in Egypt. Both host adaptation and geographic segregation would reduce the inter-species transmission of *S. scabiei* [12], [45], thus have important implications in our understanding of the epidemiology of *S. scabiei* and development of control strategies against mange in animals and scabies in humans.

The small number of specimens characterized is a major limitation of the current study. Thus, the conclusion on host-adaptation and geographic segregation in *S. scabiei* in Egypt needs support of multilocus genetic characterizations of parasites from a range of hosts, especially those of humans and companion animals. More advanced molecular biological tools, such as population genetics and comparative genomics, are also be needed to understanding the genetic mechanism responsible for host-adaptation and geographic segregation in *S. scabiei*.
